# State patients charged with parricide: a thematic analysis of their relationship experiences with their victims

**DOI:** 10.3389/fpsyt.2026.1842141

**Published:** 2026-08-21

**Authors:** Bibi F. Moosa, Navanthree Govender, Chantal Waldeck

**Affiliations:** 1 Division of Forensic Psychiatry, Department of Psychiatry, Faculty of Health Sciences, Department of Psychiatry, University of the Witwatersrand, Johannesburg, South Africa; 2 Department of Psychiatry, Sterkfontein Hospital, Krugersdorp, South Africa; 3 Department of Psychology, Sterkfontein Hospital, Krugersdorp, South Africa

**Keywords:** forensic psychiatry, mentally ill, parricide offenders, qualitative, relationship experiences, thematic analysis

## Abstract

The mentally ill offender is considered to be an independent category when discussing the typology of perpetrators. Mental illness shares many of the risk factors present in the other categories of parricide offenders; however, there has been little research into the relationship dynamics between victim and perpetrator, particularly in sub-Saharan Africa.

**Objectives:**

The objectives of this research were to explore aspects of the relationship between a mentally ill perpetrator of parricide with the victim within different contexts.

**Methods:**

This qualitative study was conducted in a forensic psychiatry setting in South Africa on six mentally ill parricide offenders. The interviews were analysed using a descriptive phenomenological approach.

**Results:**

The themes identified were closely linked with systemic injustices that resulted in the fragmentation of family structures. Inadequate resources contributed to themes of increased responsibility on younger family members and limited time for families to bond. Another closely related theme was the normalisation of substance use. Poor patterns of communication and inconsistent discipline were common to all participants. The last theme focused on the difficulties faced by parents as they struggled to understand the magnitude of mental illness in their children. The intended areas of exploration were discussed as consequences of or mediators of stress within the themes.

**Conclusion:**

The implications are for future research to focus on identifying modifiable risk factors and to explore the influence of South African culture on communication patterns in similar community settings. Of the six participants, one outlier was identified in terms of socioeconomic circumstances and the victim being a stepparent. This possibly indicates a need to clarify if relational proximity and/or social class compound risk.

## Introduction

Parricide is a category of family murder referring to the killing of one’s parent or other primary caregiver playing the role of a parent, and is divided into matricide and patricide – murder of a mother and father respectively. It is considered to be a relatively rare form of homicide (1–5). When examining the characteristics of parricide offenders, studies show a correlation between the incidence of mental illness and parricide (2–8).

### Characteristics of parricide offenders

Heide (9) divides the perpetrators of parricide into four groups: those with severe mental illness, severely abused children, individuals with antisocial personality disorder (ASPD), or the severely enraged parricide offender. (9) All these conditions have common aetiologies and risk factors, therefore the categories are blurred. Abuse, on its own, is a risk factor for mental illness and ASPD; and having mental illness makes an individual more vulnerable to abuse (10, 11).

Perpetrators are usually single or separated from their partners, middle-aged, and living with their mothers. Men are more frequent perpetrators of both patricide and matricide (7, 10, 12, 13). A study comparing criminal responsibility among males who committed parricide noted that mothers are the more likely victims of adult men with severe mental illness, while juveniles attacked their fathers, usually in response to a threat (7). Similarly, in the case of matricide, daughters were more likely to be suffering from psychosis at the time of the murder, whereas with patricide the motivation was generally abuse (8).

Parricide is committed by adults more than juveniles (1). Research suggests that juveniles use murder to act out their desire to escape abusive circumstances while the assumption is that adult perpetrators are usually mentally ill (13, 14).

### Parricide in criminal populations

Dysfunctional family dynamics are heavily implicated with emphasis on the parent-perpetrator relationship; however, this is mostly in the juvenile population and in the absence of mental illness (1, 4, 11). Common findings include various forms of severe abuse throughout childhood (15, 16).

A Brazilian quantitative study found that parent victims qualified for risky parenting styles. (11) A South African qualitative study examined nine cases of family homicide perpetrated by children and adolescents. All perpetrators had suffered abuse at the hands of their parents with extreme parenting styles. Themes of poor attachment, family dysfunction, as well as personal psychological problems, were ascertained through research findings (4).

Various quantitative and qualitative studies exploring characteristics of parricide offenders and their victims have described the victims in hostile terms. Mothers who were murdered by their sons were described as domineering, demanding, and possessive, or, in the case of daughters, dependent and hostile (17, 18). Fathers killed by their sons were described as aggressive, and both parents were frequently experienced as punitive (4, 17). Where only one parent was killed, the other was found to be absent or having played a passive role in the child’s life (4, 17, 19).

### Parricide in mentally ill populations

Menezes (20) examined parricide in Zimbabwe and found that the majority of mentally ill offenders were men, the victim population was slightly skewed towards fathers, and the most frequent diagnosis was a psychotic disorder (20). Psychotic disorders are in fact more likely to influence violence rather than mood or anxiety disorders. Those most at risk are people known to the perpetrator, which implicates proximity and relationship dynamics as contributing factors (21, 22).

In the case of adult men, mothers are the more likely victim, whereas with juveniles, fathers are more frequent targets. Studies have found that most men who committed parricide, particularly matricide, had a diagnosis of schizophrenia, whereas daughters who killed their parents were found to have a historically strained relationship with the victim although not necessarily in the context of mental illness (6–8). However, daughters who committed matricide were more likely to be suffering from psychosis at the time of the murder, whereas with patricide the motivation for murder was generally abuse (8).

Matricide is closely associated with, although not specific to, males living with schizophrenia. A Nigerian hospital produced a set of case studies that focused on two mentally ill perpetrators of matricide. The perpetrators, both of whom had pre-existing diagnoses of schizophrenia, described feelings of dependence on their mothers, although not having had close and loving relationships. The motivation for the murder was linked to delusions from active psychosis, but themes of ambivalence were observed which indicated problems with attachment in childhood. While this implicated pre-existing relationship strain and mental illness as perpetuating factors in the build-up to matricide, other aspects of the parent-child relationship were not explored (23). Similar findings were highlighted in a 2015 study that explored the motives of mentally ill matricide offenders in an Italian forensic setting. All nine participants described conflicted relationships with their mothers and absent fathers (19). These are among the few qualitative studies done on mentally ill perpetrators of parricide in forensic psychiatric settings (19, 23).

Considering the above, Miles et al. (24) suggests that the parricide is an engendered phenomenon. Patricide is not to be diminished; instead, parricide as a whole requires further understanding of the individualised contexts that lead to each incident within their cultural ecosystems. This study also described the role mothers played in taking care of their adult sons, often having to compensate for inadequacies in the healthcare system; again, implicating a dependent relationship and forced proximity as contributors to the increased risk of violence (24).

Global research is frequently set in Western societies where cultural influences on mental illness are not taken into account. The research available in African settings often references local beliefs and customs, such as witchcraft and traditional healers, as possible influences on the course of illness or motivations.(8, 20, 25–27) 

### Addressing gaps in the scientific literature

Hinde (28) suggests that the context of parent-child interactions is just as important as the interaction itself, e.g. during discipline and playtime (29). Other potentially relevant contexts pertain to the parent’s involvement in the child’s schooling and occupational life in adulthood (11). Another vital component of parenting is nurturance. When properly executed, nurturance reduces poor mental health outcomes despite the presence of external stressors (30, 31). While the consequences of mental illness have been broadly implied, there is no in-depth exploration of the patient’s experience of a shift in the relationship with the parent victim. Exploring the child’s perception of a parent coming to terms with their child’s mental illness may reveal the impact it had on their relationship (18, 23, 24, 32). The existing studies exploring parricide in mental illness are limited, primarily international and quantitative (11, 23). In African countries such as Ghana, Zimbabwe, and South Africa (SA), the focus has been on criminal populations with greater emphasis on quantifiable characteristics (8, 20, 26). There is a gap in the literature exploring the factors that perpetuate parricide in mentally ill populations, particularly with regards to the relational experiences between the perpetrator and their victim.

Further, Apartheid, a system of racial discrimination, was unique to South Africa – it tailored a strategy to restrict the rights of people of colour while creating a racial hierarchy that led to discontent between populations. While Apartheid was officially repealed in 1994, the systemic injustices remain deeply embedded in society (33). There is ample research exploring the disruptions created within families with a resulting culture of single parenthood, substance abuse, violence, and poverty; each of these feature significantly in the context of mentally ill parricide offenders (33–36). The Group Areas Act, under the Apartheid era, forced the separation of Black families. Young and middle aged men and women were forced to migrate to urban areas for work while leaving their children to be cared for by extended family or, more often than not, grandparents. Traditionally, South African culture has favoured the inclusion of the elderly in the nuclear family unit ensuring that grandparents played a supportive role in raising children. With the introduction of spatial segregation, grandparents often assumed the role of primary caregivers, forced to parent a new generation with a different set of challenges that they were ill equipped to handle. The strain on their mental health likely disrupted their ability to provide emotional support to their dependents (33). Apartheid emboldened efforts to oppress the local farm workers by compensating labour with alcohol instead of money. This was informally known as the “dop system” and it contributed to the legacy of alcohol dependence that South Africa is known for as well as contributing to mental health burdens. When present together, alcohol use and mental illness are inextricably linked with violence (37, 38). Another unique cultural phenomenon in South Africa is how spiritual beliefs influence peoples’ understanding and attitudes towards mental health. Symptoms of mental health are interpreted as spiritual manifestations that resolve with the application of traditional remedies and rituals. While the role of traditional healers is not to be diminished, mainstream interventions may ultimately be delayed where unresolved or recurrent symptoms are not recognised as mental health entities. (39) Regardless of the belief system, the stigma around mental illness remains a major obstruction to seeking help. These are all important considerations in the South African context that are not reflected in Western research.

## Objectives

The objectives of this research were to explore aspects of the relationship between a mentally ill perpetrator of parricide with the victim within different contexts: during play as a child, the parent’s approach to discipline, the parent’s involvement in schooling and later occupational life, the parent’s reaction to the onset of mental illness in the child, and the type of nurturance and support offered to the child, if any, during their times of need.

## Methods

### Study design

This study used a descriptive phenomenological approach to identify themes within the participants’ childhood experiences with their parricide victim in various interactional contexts. Data were collected by the principal researcher through semi-structured interviews with prompts based on predefined areas of interest (40–42).

### Study site and population

The study was conducted at Sterkfontein Hospital (SFH) in Krugersdorp, Gauteng, in SA. Individuals suspected of having a mental illness or intellectual disability are referred to Sterkfontein Hospital by the court, and assessed in accordance with sections 77, 78 and 79 of the Criminal Procedure Act (43). If found not fit and/or not criminally responsible, in the case of serious offences (e.g. murder and sexual offences), the person would thereafter be admitted as a State patient (SP) in terms of section 42 the Mental Health Care Act 17 of 2002 for care, treatment and rehabilitation (44).

The study population was adult State patients charged with parricide admitted to SFH. The duration of admission was not specified, provided the participant was psychiatrically stable. Should symptoms of mental illness have been present, these would not impact on the participant’s ability to meaningfully participate in the study. Other inclusion criterion were that participants could give informed consent and could converse fairly in English. As per the National Health Act all the participants were over 18 years and had no active symptoms of mental illness at the time of interview therefore they all maintained the capacity to consent to participating in the interview process (45).

### Ethical clearance

Ethical clearance was obtained from the University of the Witwatersrand’s Human Research Ethics Committee (M21/11/68). A distress protocol was created in collaboration with the Department of Psychology at SFH that ensured that any individual who required or requested it, would be seen on the same day for containment.

### Data collection

As highlighted in the literature review, parricide is relatively rare. Finding parricide offenders specifically in one region of SA who not only suffered from mental illness but were also found not responsible for the offence due to the mental illness created a particularly niche category. In order to yield the maximum number of participants, purposive sampling was employed which involved the intentional selection of specific individuals who met the inclusion criteria (46). Ten potential candidates were identified from forensic records from 2000 to 2021. Before approaching the inpatients, the researcher discussed the SPs with the ward doctors to assist in identifying SPs who might have been able to consent. Inpatients were approached in person while outpatients were contacted telephonically with four of them declining participation for personal reasons. Following a briefing by the principal researcher, agreeable candidates completed consent forms for the audio recording and use of interview material. Participants also received an information sheet providing details on the purpose of the research as well as the researcher’s role. The principal researcher had no prior established relationship with any of the participants. Five of the interviews were conducted in private interview rooms at SFH with the duration of the interviews varying based on how much information the SPs offered. One interview was conducted at a local clinic with a participant who was on leave of absence. Two interviews were repeated at the request of the SPs who had time constraints but wanted to share more. Each interview was between 40 to 60 minutes. Participant 2 terminated the interview early but did not provide a reason. The rest of the interviews were terminated once the researcher had addressed all the areas of interest and the participants were satisfied that the content they shared was representative of their experiences.

### Data analysis

The data was analysed using thematic analysis guided by the method described by Braun and Clarke. The researcher became familiar with the data; with the assistance of the software programme ATLAS.ti, frequently occurring words and phrases were highlighted or “coded.” (47). As similar codes were grouped together, themes gradually emerged and were strengthened by supporting quotes. Quotes and codes were frequently revisited in order to identify overlaps and reorganise themes and subthemes. These themes were held up against the data to measure their rigour and to define the subthemes, then, linked to the relevant domains of interest outlined in the objectives (47, 48). In order to ensure objectivity, the supervisors and principal research reviewed the data independently before corroborating their findings. The principal researcher made reflexive notes after the interviews to identify any pre-existing biases or responses that may have influenced any interpretations, and all of the themes were grounded in the data rather than the researcher’s personal views. Transcripts were not returned to the participants for corrections or feedback. To ensure transparency the researcher included as much relevant detail of the setting, population, interview technique and excerpts; this also helps the reader to determine the transferability of the data to their setting.

## Results

Six participants were interviewed for this study. They were all male with ages ranging from 29 to 38 years. Two of the participants were Black, two were Coloured, and there was one Indian and one Caucasian participant. Five of the participants were diagnosed with schizophrenia and comorbid substance use disorders. The last participant had a substance-induced psychotic disorder. The genders of the victims were evenly split - three biological mothers, two biological fathers and one stepfather.

## Themes and analysis

### Assuming adult responsibilities

The victims were raised during Apartheid, which imposed the systemic issues that dictated their harsh living circumstances. Institutional racism displaced large populations to distant areas with limited opportunities, forcing breadwinners to find low paying jobs far from home (34) admitted as a State patient (SP) in terms of section 42 the Mental Health Care. Long working hours for the victims meant that SPs had to assist with the running of the home from a young age. Early on, the SPs engaged in instrumental roles, such as caring for younger siblings and preparing family meals.

P1 recalled:” *My mother was always at work so I was owning the house … I was in grade 1…I would fix my lunchbox … I could cook… maize, rice, eggs … I would make sure that everything is ready*

*[before] she comes back from work…* “

P1: “It was when my younger sister wanted to drink paraffin … I saw her from a distance so I came running trying to stop her … my mother was at work…”

Another consequence the SPs suffered due to their poor finances was the pressure to contribute to the household. Without the appropriate support, some SPs left school to find work and, in some cases, became the primary breadwinner.

P1 spoke of his mother’s struggle to support the family: *“…the stress that my mother can no longer buy the stock, can no longer make money so then in 2011, I failed my matric. From there, I went hustling.”*

Some of the SPs were slightly more privileged but still contributed where they could.

P2: “I used to [financially] support [my father] because I knew he was going through a hard time.”

P4: “I was always giving him something like money … I was even bringing [food] hampers from the workplace … he was suffering.”

P3: “I got a job driving taxis. I told myself that she won’t suffer … I’d look at her budget first … if she doesn’t have, I’ll give her maybe R400.”

Parentification is when children assume adult responsibilities such as described above – it is frequently referenced in studies exploring poverty and trauma as mediators of adult mental health (49–51). Research on parricide in the general population implicates circumstances of severe deprivation (11, 14).

### Lack of play during time together

Parents’ work obligations and financial stress curtailed important bonding time, such as (but not limited to) playing together. Regarding playtime with parents, the SPs instead described various non-play activities that they did alongside their parents. Some activities were passive:

P2: “I never played games with my father but we would usually do jobs like maybe weekend[s] … I’d be with him doing things like construction … we’d be watching our TV but he’ll be doing his own thing.”

Other activities were more practical such as performing chores or work-related activities:

P5: “I was helping my mother clean the house. I would help her wash the windows … do the washing … I used to enjoy her cooking and ask her [how she makes things]. So, I used to be in the kitchen with her a lot.”

P4: “[We were] finding jobs [for] me and my dad.”

Playful interactions with the victims were rare and mostly occurred in early childhood:

P1: “My mother used to put me on top of her legs, swing me up and down … ask me about creche … It was [not something for everyday] because she was working.”

P4: “He [bought] play things for me … soccer ball … Barbie dolls [for my sister] … we were still young … [We used to] watch movies together as a family.”

More often than not, playing or socialising was done separately.

P1: “It wasn’t in such a way [where] I could play with my parents … I normally played with my friends in the streets.”

P5: “…kids must be on their own, adults on their own…”

Studies exploring the association between poverty and parenting styles indicate that parents in lower socioeconomic circumstances are focused on survival and may therefore engage less meaningfully with their children. For some of the SPs an additional barrier was noted when parents worked away from home and relied on grandparents or extended family as caregivers. This resulted in lost opportunities to establish understanding and trust in the parent-child relationship, and later impacts mental health for adult children (52–55). As illustrated by the literature, this strain also extends to the substitute caregivers who may not be equipped to manage the emotional needs of the children they foster (33, 54).

### The normalisation of substances in the living environment

When the SPs’ spoke about their parents socialising with others, alcohol almost always featured as a focal point in these gatherings. P3 spoke with amusement of his mother’s behaviour while intoxicated:

“At that time I didn’t know what was drunk. She slipped and she started vomiting, so I took a bucket, I [cleaned up] and gave her something … when we woke up in the morning she said “who cleaned [up]?” I said it’s me, and then she said “you love me, huh?” Even when I was grown up, she used to talk about that story, “you remember that day I was drunk?”“

The victims’ relationships with alcohol arose organically and unusual situations were discussed seemingly casually.

P2: “[My father] would drink on the job and come home and still drink … whenever he had the chance of drinking he drank … [a] lot of people [sold] alcohol from home so we would go around and ask for a credit here and a credit there… “

P3 grew up with easy access to alcohol. He described road trips with his mother where “ *she would ask me if I have some cigarettes … do you have money for alcohol? Then they will give me money and I will go enjoy.”*

P3: “[My mother and her friends] would call [us kids] and say, ‘you don’t want to drink that cool drink?’ Then they gave us the alcohol [and] we drank.”

P4: “[My father] was a violent person when he became drunk. [He used to] push us around the house … [fought] with my mom.”

P1 did not discuss his mother’s relationship with substances, however his exposure to substances stemmed from home:

“Sometimes, my father would come home drunk … he would water the yard and sweep and tell everybody in the house no one must get out of the house.”

Patel (38) explained the context of alcoholism in South Africa as a product of Apartheid practices. Poor Black labourers were given alcohol as payment while socioeconomic resources were misappropriated away from marginalised communities. This led to a legacy of alcohol consumption as a social activity and a way to cope with stress and trauma (38). All of the SPs in this study had significant histories of substance use long before the onset of their psychotic illnesses, particularly alcohol and cannabis. Cannabis use appeared to be seldom addressed by parents. This could possibly be explained by literature examining attitudes towards substance use in

SA which found that cannabis was the least stigmatised substance and that cannabis users were considered comparatively less dangerous to other substance users (56).

The SPs’ cannabis use likely facilitated the onset of mental illness (57–59).

### Limited communication within the family

Limited communication comprised four sub-themes. The first subtheme is about the difficulties relating to each other particularly when it came to parental involvement in the SPs’ schooling. Indirect expressions of affection discuss the alternative ways that families communicated their caring. Lastly, cultural hindrances that led to the avoidance of difficult conversations underscore the tacitly understood and deeply ingrained social norms that were prominent in the SPs from non-White families. Difficulty relating to each other P2 described a shared experience amongst the children in his community, *“…I don’t think the parents were really involved. Like we [school going children in the neighbourhood] all just meet in the street and we all walk up.”* Communication between SPs and victims was experienced as closed off for various reasons. Parents’ limited education made it difficult for them to relate to the pressures of school.

P3: “…she didn’t go to school so it was difficult for her to maybe help me with my homework … we didn’t talk about school things.”

Despite having a close relationship with his mother who didn’t attend school, P5 struggled to articulate his academic difficulties to his mother for fear of disappointing her:

“I remember feeling a bit trapped … I did not show her anything cause; I did not want her to know … I did not want her to think that I am not working at school…”

### Indirect expressions of affection

Victims were, however, sometimes able to express their affection in different ways.

P3: “[My mother wouldn’t] say “shap” (sharp), give me hugs, things like that … She [made sure there was pap (food) every day], [she] used to give me the drumstick and the shoulder of the chicken. [She] knew it was my favourite.”

P4: “[My father] was always there for me … in the back of the yard, he built a room for me … he was always there for our small children … he was mad for them.”

SPs proudly described the skills their parents had taught them as a way of ensuring their success.

P2: “ Honestly speaking his actions did show concern like what must I do … like be something in life … When we were doing a job together … he did show me how things must be done and showed me the proper manner.”

P1: “[My mother] took care of me, she taught me how to cook, take care of myself, how to be responsible and she taught me to respect others…”

Overt affection was sometimes described in early childhood, such as with P1 and P4 experiencing physical affection from their mothers. A 2022 article reviewing the literature on the cross-cultural similarities and differences in various aspects of parenting highlighted how non-Western cultures displayed less “warmth” towards their children (60). Instead, warmth is demonstrated through actionable services.

Some actions were less direct but nonetheless interpreted as affection:

P4: “ Then me and [my] girlfriend [broke] up … But my father always go there to check up on [my] child.”

P5: “ She used to … like always … giving advice … telling me things to say what I must do. How I must carry myself, what I must do at home, how I must act… “

The above may serve as evidence that the victims found ways to circumvent direct communication of positive feelings.

### Cultural hindrances leading to the avoidance of difficult conversations

Serious conversations about death and feelings were rare. For P3, the avoidance of emotionally charged discussions was reinforced by a hyper-masculine culture that discouraged asking for help (57, 61).

P3: “When you go to an initiation school … they teach you that no more mummy now, you’re on your own…”

When P3’s brother was murdered, they “*never spoke about it*.” When his mother made puzzling comments about the incident P3 didn’t pursue it:

“I didn’t ask her why she is saying that … In our culture, when an elder says something, you cannot say they are lying.”

Factors such as age gap and culture influence discussions about sensitive topics. Qualitative studies based on South African populations describe a sense of disapproval held by caregivers when it comes to sex. It is postulated that in the face of deprivation, respectability is clung to as a source of power, and sex does not fall in line with these standards (62, 63). When it came to relationships, P1 described his mother as “ *old school*.” In turn, the SPs reflexively shied away from communicating with their parents, sometimes out of fear of repercussions.

P1: “I was also afraid of her to be honest … she was strict and scary. I couldn’t [make] eye contact when I was chatting with my mother.”

“[She was one of] those people who are not comfortable talking about relationships with children … she didn’t want me to get involved in relationships with girls.”

P2: “We’re not people that I can say we’re open to one another … like … at the end of the day, we wouldn’t express our feelings”

P4: “ When he is sad or something, I cannot talk to him … he will get cross with me.”

P1 was unable to approach his mother about certain topics, even when their safety was at risk:

“I never thought that it could go to a place whereby he grabs her with his hands and beat her up because he did that and she didn’t open a case against him … sometimes as a child you cannot interfere [in] adults’ relationships…”

“Normally we slept in the same room. It was me, my mother and my sister … that other day I saw my sister sleeping on the floor when I woke up … I greeted my mother, she introduced me to my stepfather in the bed … we can’t sleep with a stranger in the same room. She didn’t think for us.”

In the process of describing what the SPs viewed as mostly unremarkable childhood memories, patterns of physical abuse, exposure to harmful substances, and poor supervision emerged. Despite this, the SPs sometimes appeared to unknowingly contradict themselves, such as P4 who described his father as *“always there for us”* and his drinking as *“not that much”*, yet described his father *“going to his girlfriend”* and *“[coming] back drunk.”* Similarly, P1 described his mother being *“loving”* and “ *[taking] care of [him]”* yet also described feeling *“scared”* and as if they *“never bond[ed].”* The culture of deference to elders likely contributed to this attitude, but a more complex psychological defence, like splitting may be implicated (51). This pattern of avoidance exposes the lack of direct communication about important topics such as sexual conduct, trauma, and, by extension, harmful behaviours.

### Inconsistent discipline

In keeping with the previous sub-theme of avoiding difficult conversations, the SPs described opportunities for guidance or discipline that their parents had missed.

P1: “She wasn’t interested about maybe asking me as her child, ‘why are you [smoking cannabis]? Can’t you see that you’re ruining your life?’ She didn’t care no more. So I continued using cannabis until I hurt myself…”

A pattern of poor communication combined with high levels of stress possibly limited parental capacity to confront the SPs’ about their conduct (52, 54, 55).

P3 related multiple incidents during early adulthood where he got into trouble while intoxicated and his mother would come to his rescue. At the time,

P3 recalled: *“My discipline was don’t drink at a shebeen, if you need alcohol go and buy it and come back home … at the tavern you [might] stab someone … [whereas] if you’re at home they’ll say “ah man, you are drunk! Go and sleep!”*

P3 later reflected: “ *I can say she didn’t guide me. [She didn’t tell me] the consequences of dagga, alcohol … It’s like I was on my own.”*

The consequences of poor communication also included a lack of direction on a day -to- day basis.

P2: “ He would talk about cleaning the yard and things but at the end of the day nobody knew who’s supposed to clean the yard … the yard needed to be cleaned but he never said who must clean it. “

Corporal punishment was frequently reported to have been enforced as a form of discipline, and SPs felt that not all of the behaviours described warranted severe consequences.

P1: “Whenever she found me with a girl on the street she would shout. One day, I was with my girlfriend inside the house [and] she caught us there … she went to the tree, broke a stick, and … she beat us up.”

P3: “I don’t remember what I did to my mum and my mum said to my big brother, go and take a stick so that I can beat these young children.”

P2 described the discrepancy in his discipline in a more emotionally distanced tone:

“He would go to work and come back and expect us to do our chores … sometimes he does like, give us a hiding if we do not do our chores and things.”

“Me and my friend … we lifted this girl’s skirt and that girl reported us and they expelled us from school … [my father] never said anything … we never spoke, not like that. He did say

‘why you do that’ but not fighting about it.”

P4 described his experiences in a similar tone:

“Maybe me and my sister was fighting. Then he would take the belt and … hit us.”

When P4 decided to leave school his father was unresisting in his response:

“Then I told him [father] I’m getting tired of school now. Too much hard work. [He said] you are an adult. I cannot protect you for the rest of your life. You have to learn your lesson and maybe see how life is going to treat you.”

P5 also experienced corporal punishment; however when his mother learnt of his substance misuse she facilitated his admission to a rehabilitation facility.

Parental alcoholism, poverty and high levels of parental stress seemed to result in the adoption of harsher parenting practices. Corporal punishment in particular was a legacy left from Apartheid (64). It is more reflective of parental emotional state and a precursor to child abuse rather than an effective form of discipline and, much like authoritarian parenting, it secures short term compliance rather than builds internal motivation to change behaviours. It is now classified as a form of structural violence rather than a method of discipline, with strong evidence to support the harm that it causes; it erodes children’s trust in their caregivers and normalises violence as a part of conflict resolution (35, 65, 66).

### Parents’ difficulties in understanding mental illness

For the SPs who received their diagnosis before the incident, parents appeared to struggle to understand the significance of a mental illness diagnosis. The victims supported the SPs through conventional treatment, but also sought alternative care from traditional healers.

P1: “[My mother] is the one who took me to the traditional healer … I think due to lack of information, she also thought that I was bewitched.”

P3: “…they were running around, going to sangomas, wasting money to make me get better.”

This is a common practice in South African cultures and is valuable when appropriately applied (67). A 2023 study based in Gauteng, SA examined the pathways to care for mental illness and found that almost half of the study population visited one or more traditional healers multiple times before presenting to a healthcare facility. This finding was in keeping with similar studies based in sub-Saharan Africa. Opting for alternative methods of appropriate treatment for mental illness may delay its timeous management. This delay means that patients endure a longer period of untreated psychotic symptoms. In cases where the patient is aggressive or driven by persecutory delusions, family members’ close proximity increases their risk of adverse events, such as physical harm (68–70).

That being said, the deficiencies within the public health system in SA leave families with limited options. Stigma and a lack of knowledge regarding mental health further add to the obstacles of seeking help (57, 71).

P5 had not been diagnosed yet but recalled his mother’s reaction to his symptoms: “[My mother] used to tell me sometimes you know, just stop listening to what is going on in your head … I could not get her to understand what I was going through … with regards to the schizophrenia and voices in my head.”

At the time, P5’s mother attributed his symptoms to substance use alone. She facilitated an admission to a rehabilitation facility. However, P5 likely would have benefitted from a psychiatric admission. This speaks to a wider challenge regarding mental health literacy (71).

P4 was diagnosed after his father’s death but in the days leading up to it, he spoke mainly of his father’s excessive drinking and violence.

## Outlier

P6 had similar experiences with abuse and exposure to substances. However, unlike the other SPs, P6 was Caucasian, and he grew up in an affluent family. While the other SPs left school in their late teens to take care of their families, P6 completed high school and his stepfather *“bought [him his] first car [and] paid for college [and an] apartment.”*

Similar to biological parent-child relationships, stepchildren whose stepparents employ harsh disciplinary methods and an authoritarian style of parenting are more likely to share conflicted and emotionally distant relationships (72). P6’s stepfather *“took over his discipline”* when P6 turned 11 years old, and P6 recalled his treatment as *“extreme mental abuse”*. P6’s mother tried to *“protect”* P6 by *“defending [him]”* and *“[hiding] a lot of stuff from [his stepfather]”* which, according to the available research, may have adversely affected the relationship between P6 and his stepfather (72).

A younger age of introduction, positive attitudes from the biological parents, and mutual efforts from stepparents and stepchildren to connect with each other encourage parent-like relationships (72). P6 was introduced to his stepfather when he was eight years old. At first, they *“got on alright.”* He felt like his stepfather *“was a really interesting person, intelligent”* and *“[P6] really wanted to be his friend,”* yet he recalled distressing interactions between his mother and stepfather:

“I saw how he treated my mother, [it] brought fear into me.”

Other contradictions included P6 recalling his stepfather as “kind” with “a good heart,” yet also described him as “evil” and “[having] some hating towards [P6].” P6’s stepfather “gave [him] opportunities … two or three jobs,” but “always found a reason to fire [him].”

Research on the evolution of stepparent and stepchild relationships proposes that stepchildren changing their opinions of their stepparents is a recognisable pattern of relationship development. (72) P6 may also have been influenced by the abuse his stepfather inflicted on his mother:

“[P6’s stepfather] is busy strangling my mother. My mom’s screaming to me to call the police … I’m young, I can’t do anything about it.”

“She told me many times, I wish he would just die…”

A biopsychosocial framework provides an understanding of the different factors that determine mental health. Specifically in the context of this research the psychological and social aspects provide background that helps to conceptualise the SPs. The socioeconomic adversity, allocation of healthcare services, as well as the fragmented family system were typical for people living in South Africa under Apartheid. In many ways, the parenting experienced by the SPs was a response to circumstances driven by culture and authority, and to some extent, society. Being confronted with severe socioeconomic adversity shifted the focus of families from thriving to survival, a concept expanded on in Maslow’s hierarchy of needs (73). Beyond the physiological needs, parents strove for safety needs with fluctuating financial security. Apart from corporal punishment, the SPs were mostly physically safe and healthy. Levels of love and belongingness varied amongst the SPs. Static and dynamic factors such as parental personality, gender, and substance use, amongst others, influenced parental affection and nurturing. A common element of material deprivation found within the themes compounded the childhood experiences described above. Esteem needs were seldom attained by the SPs who acquired a similar survival mindset from their parents. The limitations of Maslow’s hierarchy of needs are addressed broadly in sociological frameworks where distressing experiences are believed to create the scaffolding for mental illness. In circumstances where the distressing experience is a way of life, such as with poverty or abuse, people are more likely to struggle with poor mental health outcomes (74).

## Biases and limitations

All of the SPs were fluent in English; however, for three of them, their first language was the local dialect. This may have compromised their ability to express themselves fully. The protracted history of psychosis and substance use may have impacted cognitive function which resulted in some stilted conversations where the researcher struggled to extract more meaningful data. The researcher distinguished their role from that of the managing multi-disciplinary team in an attempt to limit acquiescence bias, however, it cannot be eliminated altogether. The SPs’ memories were possibly influenced by guilt or protective feelings towards their parents. They may have also struggled with feelings of ambivalence and employed a protective mechanism, much like splitting, which may have led to recall bias.

## Conclusion and implications for future research

The aim of this study was to explore the relational experiences of parricide offenders with their parents from the SPs’ point of view. Although mental illness is acknowledged, it is not labelled as the sole determinant of risk. A key determinant to the SPs’ relationship experiences with the victims was the inception of Apartheid which fractured family units permanently and enforced a cycle of disempowerment and maladaptive coping mechanisms that continues to pervade underprivileged communities in South Africa today. An authoritarian style of parenting was favoured in the face of stress because it established the parent as the one in charge and achieved desirable behaviours in the short term. In actual fact, relationship quality was compromised and important messages were not conveyed. Substance use and mental illness intensified the existing struggles. Four of the victims functioned as single parents. Available research has not necessarily highlighted single parenthood as a characteristic of parricide victims; however, its presence imparted significant disadvantages to the SPs affected. Single parenting is a common reality in South Africa and considering its negative impact on social development and behaviour, is worth exploring as a potential area to provide support (75, 76).

The discussion of P6 implied a different dynamic between children and stepparents who fall victim to parricide. Bearing in mind that P6 came from relative wealth, further explorations can possibly clarify if stepparents fall victim for similar reasons or if social class alone accounts for the variables.

Future research will hopefully apply similar qualitative methods in the forensic population in order to highlight their experiences and identify modifiable risk factors. For the time being, the impact of culture and hyper-masculinity on communication amongst South African men in lower socioeconomic populations may highlight some of the challenges they face. Future researchers may also consider exploring these patterns of communication further in order to understand the origins and significance within the culture. In doing so, there may be a way to circumvent communication limitations without infringing on important cultural practices.

Existing risk factors for mental illness and strained parent child relationships, such as parenting style, have been explored well enough to initiate actionable change. A 2025 publication put forward a comprehensive parenting framework within the context of a post-Apartheid South Africa. The key suggestions were to augment support structures with the assistance of specialised daycare and aftercare services, community involvement, and parental training on effective parenting practices (64). These efforts may have benefitted the State patients. Changing policies should address ongoing challenges such as limited access to healthcare and the unsafe use of substances, and realistic solutions must be implemented specifically to improve mental health literacy in the community.

A notable similarity between some of the SPs was the tendency to lean into their culture to seek answers. In SA, traditional healers are recognised entities as dictated by the Traditional Health Practitioner Act 2003 (77). As suggested by the South African Society of Psychiatrists (SASOP), it makes sense to invite traditional healers to participate in the screening and early intervention of mental illnesses since they are the inevitable first port of call for community members in distress (78). A 2025 publication elaborates on the importance of effective governance to facilitate collaborations between traditional health practitioners and primary healthcare providers in the mental health services (79). A collaboration has the potential to improve mental health literacy and diminish the stigma attached to mental illness and people who seek mental health care.

## Data Availability

The raw data supporting the conclusions of this article will be made available by the authors, without undue reservation.
